# Noise level in a neonatal intensive care unit in Santa Marta - Colombia.

**DOI:** 10.25100/cm.v48i3.2173

**Published:** 2017-09-30

**Authors:** Angélica Patricia Garrido Galindo, Yiniva Camargo Caicedo, Andres M Velez-Pereira

**Affiliations:** 1 Research Group on Environmental Systems Modeling, Environmental and Sanitary Engineering Program, Universidad del Magdalena, Santa Marta, Colombia.

**Keywords:** Critical Care, Noise Pollution, Benchmark Standard, Neonatal Intensive Care, Environmental Health, Cuidados críticos, contaminación Sonora, estándares de referencia, cuidado intensivo Neonatal, Salud Ambiental

## Abstract

**Introduction:**

The environment of neonatal intensive care units is influenced by numerous sources of noise emission, which contribute to raise the noise levels, and may cause hearing impairment and other physiological and psychological changes on the newborn, as well as problems with care staff.

**Objective:**

To evaluate the level and sources of noise in the neonatal intensive care unit.

**Methods:**

Sampled for 20 consecutive days every 60 seconds in A-weighting curves and fast mode with a Type I sound level meter. Recorded the average, maximum and minimum, and the 10th, 50th and 90th percentiles. The values are integrated into hours and work shift, and studied by analysis of variance. The sources were characterized in thirds of octaves.

**Results:**

The average level was 64.00 ±3.62 dB(A), with maximum of 76.04 ±5.73 dB(A), minimum of 54.84 ±2.61dB(A), and background noise of 57.95 ±2.83 dB(A). We found four sources with levels between 16.8-63.3 dB(A). Statistical analysis showed significant differences between the hours and work shift, with higher values in the early hours of the day.

**Conclusion:**

The values presented exceed the standards suggested by several organizations. The sources identified and measured recorded high values in low frequencies.

## Resumen

**Introducción:**

El ambiente de las unidades de cuidado intensivo neonatal está influenciado por numerosas fuentes de emisión de ruido, que contribuyen a elevar los niveles de ruido y que pueden provocar deficiencias auditivas entre otras alteraciones fisiológicas y psicológicas sobre el neonato, así como problemas al personal asistencial cuando se exceden los niveles.

**Objetivo:**

Evaluar el nivel y fuentes de emisión del ruido en la unidad de cuidado intensivo neonatal.

**Métodos:**

Se muestreo durante 20 días continuos cada 60 segundos en escala de ponderación frecuencias A y espacial *fast*, con un sonómetro tipo I, se registraron los parámetros acústicos de nivel medio, máximo y mínimo, y percentil 10, 50 y 90. Se integraron en periodos horarios y por turno, y se estudiaron mediante un análisis de varianza. Las fuentes se caracterizaron en tercios de octavas.

**Resultados:**

El nivel medio reportado fue 64.00 ±3.62 dB(A), con máximo de 76.04 ±5.73 dB(A), mínimo de 54.84 ±2.61 dB(A) y ruido de fondo de 57.95 ±2.83 dB(A). Se identificaron cuatro fuentes con rango entre 16.8-63.3 dB(A). El análisis estadístico mostró diferencias significativas entre una media y otra con valores mayores en las primeras horas del día.

**Conclusión:**

Los valores presentados superan los estándares sugerido por diversas organizaciones. Las fuentes identificadas y medidas registraron mayor aporte en las frecuencias bajas.

## Introduction 

In the environment of the intensive care units, auditory stimuli for the newborns occur daily, frequently associated with the noise caused by the alarms from the medical equipment, telephones, conversations among personnel, closing and opening of doors, and things falling within the unit ^1^. 

These stimuli caused by noise produce four types of adverse effects on newborns, especially among premature newborns, such as somatic effects, sleep disturbances, auditory damage and problems in their emotional development ^1^ ^,^ ^2^, as well as the possible repercussions among the care staff ^3^. Brown stated that excessive auditory stimulation creates negative physiological responses, such as sleep apnea and fluctuations in cardiac frequency, blood pressure and oxygen intake^4^. It was estimated that noise from voices and monitor alarms can generate an increase in the level of noise around 120 A-weighted decibels, also dB(A), in the units, ^5^ causing hearing loss, alterations in the newborn's development, irritability, stress and negative effects on the development of the newborn's sensory nervous system ^6^.

Studies have been published that show the average levels of noise in the neonatal intensive care units (NICU). In Chile, values between 45-80 dB(A) were obtained ^2^; in Lima (Peru), between 62-76 dB(A) ^7^ ^,^ ^8^; in Madrid and Huelva (Spain), between 51-88 ^9^ ^,^ ^10^; in Tabriz (Iran), between 56-70 dB(A) ^6^; and between 58-70 dB(A) from other studies ^8^. These are high values when compared with the limits stipulated by the World Health Organization (WHO) that establishes values of 35 dB(A) for the day and 30 dB(A) for the night ^11^.

In the Colombian Caribbean region, there is a lack of published studies related to the subject of verifying the noise levels that are managed inside a NICU in the region, which is why the equivalent continuous noise levels were assessed in Santa Marta in a hospital's neonatal intensive care unit.

## Materials and Methods

The study was carried out in the neonatal intensive care unit (NICU) of a hospital located in Santa Marta (Colombia), in a public high complexity medical center in the region, which provides medical services while teaching university students in the health area.

The unit has fourteen beds authorized for neonatal critical care, with an average occupation rate of 58% (8±1 per bed) and 5±1 availability of care staff ^12^ on three shifts: morning (07:00-13:00), afternoon (13:00-19:00) and night (19:00-07:00^+1)^. Furthermore, there are eight feeding schedules per day in 3 hour periods, beginning at 00:00. Finally, in the NICU, there are two, 20 min visiting periods at 11:00 and 17:00.

### Sampling design

#### Noise level in the NICU

The sampling was done continuously for 20 days in the NICU, considering the methodology laid out in the study developed by Vélez-Pereira ^13^ and Fortes-Garrido et al ^10^. A Casella type 1 sound level meter, CEL-633-C1K1 model, was used, and it was programmed to record data every 60 seconds using the A frequency weighting filters and the Fast temporary weighting filter. The sound level meter was located in the intensive care unit, taking into account the study developed by Vélez-Pereira ^13^, the internal dynamic of the ICU and the conditions provided by the care staff coordinators. The sampling point was located in the environment of the unit at a distance of 60 cm from the ceiling and 215 cm from the wall.

The acoustic parameters recorded were the A-weighted equivalent continuous level (LAeq), the maximum A-weighted level (LAmax) and the minimum A-weighted sound level (LAmin), in order to analyze the temporary variation of noise in the Unit. Additionally, the 90th percentile acoustic parameters (LA_90_) were recorded to establish the background noise of the unit, the 10th percentile (LA_10_) to establish the dynamic of the peak times or random noises, and the 50th percentile as a contrast of the noise in the unit, which is associated with the existing dynamic among the peak and bottom values of the unit.

#### Sources of noise emission

A visual identification of the possible sources of emission was done, and then the sound level meter was programmed and a spectral analysis was performed in one-third octave bands without a frequency weighting filter (flat frequencies), which allowed to determine each band's contribution to the noise levels. The measurement was done within the unit under the influence of other sources of noise emission, justifying the immobility of the equipment mainly due to the dynamic and demand of cubicles in the intensive care unit. The measurements were carried out at a distance of 1.35 m from the sources and at 1.20 m from the level of the floor during three minutes. The same sound level meter, which was used to measure the NICU's environmental noise, was used.

### Processing the Information

#### Equivalent continuous level of noise in the unit

The Integration of the data recorded of all the noise parameters was done in Microsoft Excel® for two periods, the first corresponding to the care staff shifts (morning, afternoon and night) and the second corresponding to the hourly values. These integration periods were established to determine the hourly variation of the noise throughout the day and the shifts, establishing the possible influence in the dynamics of the noise level in the NICU.

The integrations were done following equation 1. 



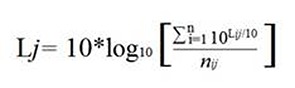
Equation 1


 Where *j* corresponds to the integrated acoustic parameter, while *i* corresponds to the number of observations or measurements taken in the integration time interval (hour or shift) and *n* is the total information observed for the acoustic parameter *j* in time interval *i*.

### Statistical Analysis

The statistical analysis was executed with the IBM SPSS 20 program. An ANOVA^6^,^13^ was performed to compare the averages of the different integration times. Furthermore, the information is confirmed through the Spearman correspondence analysis. Finally, a correspondence analysis was done between the two groups of acoustic parameters (LAeq vs LA_50_, LAmax vs LA_10_ and LAmin vs LA_90_), in order to verify the feasibility of the obtained records.

## Results

**Figure 1:**
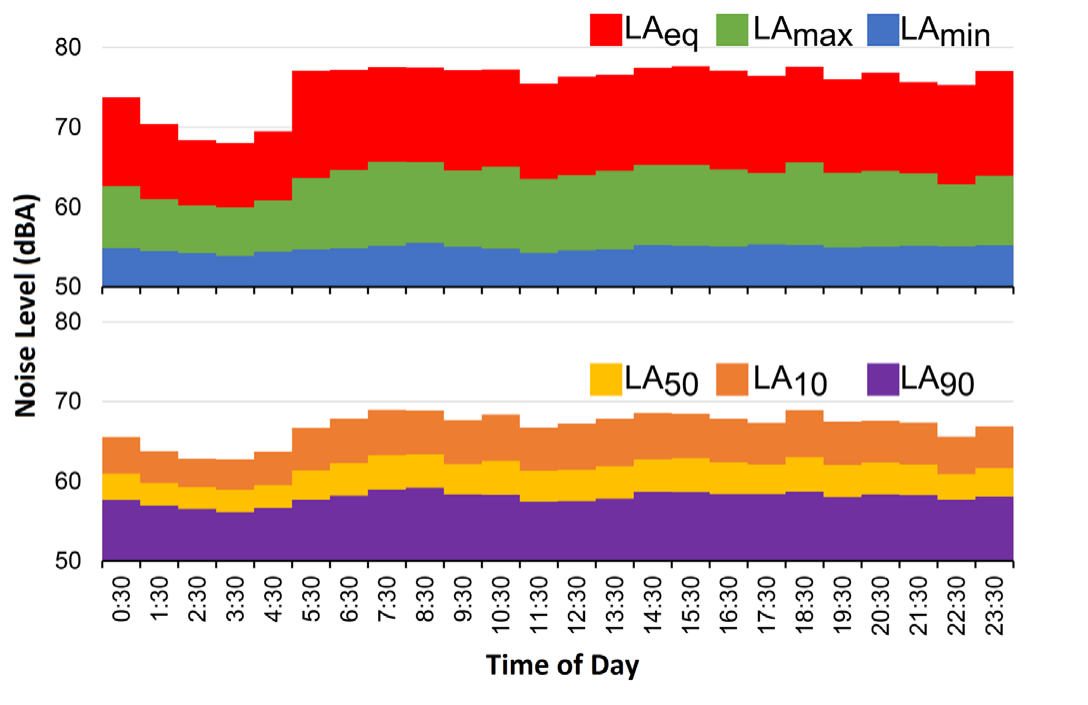
Average level of noise throughout one sampling day for the acoustic parameters. Above: LAeq, LAmax and LAmin; below: percentiles LA_50_, LA_10_ and LA_90_.

The hourly LAeq values showed average values between 59.54 ±0.50 dB(A) for 03:00 and 65.27 ±0.46 dB(A) for 08:00, with a maximum value of 69.96 dB(A) (14:00) and a minimum of 57.80 dB(A) (03:00). In Figure 1 (above), the variation of the middle hours of the day can be observed. There, it can be seen that in the early hours of the day (23:00-5:00), the values of the equivalent continuous level of noise were much lower than those presented in the other times.

For the LAmax, values were reported in a range of 67.22 ±3.01 dB(A) (03:00) and 77.32 ±1.52 dB(A) (07:00), with a maximum value of 83.70 dB(A) (23:00) and a minimum value of 59.73 dB(A) (03:00). Finally, for the case of the LAmin, the values varied between 53.16 ±2.45 dB(A) (03:00) and 55.08 ±1.88 dB(A) (08:00), with a maximum and minimum of 61.23 dB(A) and 49.83 dB(A), respectively. The dynamic of these last two parameters was similar to LAeq (Fig. 1, above); notwithstanding, it was seen that the variation of the levels of daily noise was slightly greater in the LAmax values, followed by LAeq and LAmin. 

**Figure 2:**
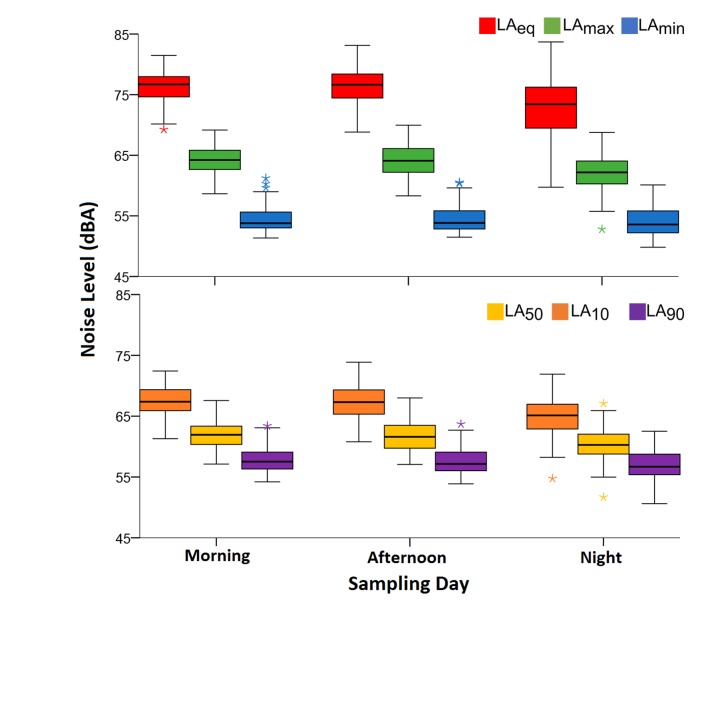
Average level of noise by sampling day for the acoustic parameters. Above: LAeq, LAmax and LAmin; below: percentiles LA_50_, LA_10_ and LA_90_.

On the other hand, the noise levels by shift showed that LAeq varied between 64.73 ±1.43 dB(A) and 63.01 ±1.45 dB(A), with a maximum of 68.05 dB(A) and a minimum of 60.27 dB(A). Subsequently for the case of LAmax, values in a range of 74.71 ±1.46 dB(A) to 76.99 ±1.41 dB(A) were present, with maximums and minimums of 80.57 dB(A) and 71.56 dB(A), respectively. Finally, the LAmin varied between 50.54 ±2.1 dB(A) and 54.65 ±1.7 dB(A), with a maximum of 59.58 dB(A) and a minimum of 51.45 dB(A). The maximum values of these ranges in the three parameters were present in the morning shift, the minimums in the night shift, while the absolute maximums and minimums were present in the afternoon, except for the minimum LAmin, which was reported in the night shift (Fig. 2, above). Therefore, it can be affirmed that the acoustic parameters declined in the middle of the night (night shift) and increased in the morning and afternoon shifts. This dynamic was similar to the hourly averages with fewer changes (Fig. 1, above).

The LA_10_, LA_50_ and LA_90_ acoustic parameters showed that the average times of peak noise (LA_10_) varied between 62.25±2.39 dB(A) (03:00) and 68.58±1.69 dB(A) (18:00), with a maximum of 73.87 dB(A) and a minimum of 54.76 dB(A). The LA_50_ varied between 58.39±2.35 dB(A) at 03:00 and 62.85±2.02 dB(A) at 08:00, with a maximum and minimum of 58.00 dB(A) and 51.69 dB(A), respectively. The background noise (LA_90_) varied less than the two previous parameters (55.51±2.42 dB(A) at 03:00 - 58.74±1.87 dB(A) at 08:00), with a maximum of 63.70 dB(A) and a minimum of 50.61 dB(A) (Fig. 1, below). The hourly fluctuations of the percentiles show that from 22:00 to 04:00 there was a progressive decline, which increased at 05:00 and remained stable for the rest of the day. In terms of the averages by shift (Fig. 2, below), a major variation was observed in LA_10_ (range 67.94±1.47 - 66.85±1.41 dB(A), maximum 71.31 dB(A), minimum 63.06 dB(A)), followed by LA_50_ (range 62.30±1.53 - 61.00 ±1.18 dB(A), maximum 65.91 dB(A), minimum 58.64 dB(A)), and ending with LA_90_ as the most stable parameter (range 58.18 ±1.61 - 57.39 ±1.81 dB(A), maximum 61.88 dB(A), minimum 54.36 dB(A)).

**Figure 3:**
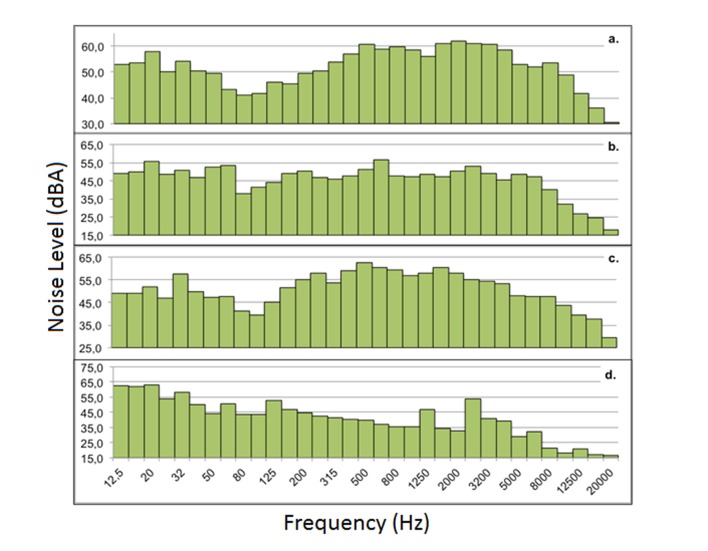
Spectrum of the studied noise sources. Results from the spectrum of a) A1 sign monitor, b) A2 sign monitor, c) A3 sign monitor, d) telephone

In terms of the emission sources, four sources were identified and characterized, three of which were vital sign monitors of different brands and models and the telephone used for communication (Fig. 3). In general, the results showed similar contributions in the different one-third octave bands. Where the alarms from monitors 1 and 3 had less of a contribution in the low frequencies (from 0 to 125 Hz), while the alarm from monitor 2 and the telephone showed less contribution in high frequencies (greater than 500 Hz).

Promptly, the alarm from monitor 1 varied between 30.6-62.1 dB with a standard deviation of 7.8 dB, presenting a greater value in the high frequencies with an average of 58.09 dB, followed by the middle frequencies (160-400 Hz), with an average of 52.9 dB, and finally with the low frequencies of 51.86 dB (Fig. 3a). This same dynamic was present with the alarm from monitor 3 (Fig. 3c), with a similar variation range (29.3-62.7 dB, standard deviation 7.7 dB), as well as the support by frequencies (high frequencies: 56.65 dB, middle: 56.34 dB, low: 50.46 dB). The alarm from the second monitor (Fig. 3b) contributed more in the low frequencies (50.55dB), followed by the high frequencies (49.20 dB) and middle frequencies (48.36 dB), varying in a range of 17.7-56.6 dB and a standard deviation of 8.8 dB. The telephone spectrum showed an order of frequencies by contribution of low (57.92 dB), middle (43.79 dB) and high (42.91 dB), with a greater variation range (16.8-63.3 dB) and higher standard deviation (12.9 dB). In this last source, three tones marked on three one-third octave bands of a central value of 1,250 and 2,500 Hz were seen (Fig. 3d).

The analysis of variance showed a significant difference between one hourly average and another, with a significance level of 99% for the LAeq and LAmax, while for the LAmin, it did not. In contrast with the ANOVA, the Spearman coefficients confirmed the information, showing a significant relationship with *p* <0.01 between the hourly averages of LAmax and LAeq, while LAmin showed a slight relationship with *p*<0.05 (Table 1). These results were similar to the averages by shift, only the Spearman correlation coefficient established an indirect and significant relationship for LAeq and LAmax, and an insignificant relationship for LAmin (Table 1).

**Table 1:** Analysis of Variance of the noise levels in the NICU of the hospital.

Acoustic Parameter	Statistical		Hourly Integration			Shift Integration		
	x̄	*S*	F	P**	SC	F	p**	SC
LAeq	64.00	3.62	10.94	0.00	0.269**	16.44	0.01	-0.381**
LAmax	76.04	5.73	25.04	0.00	0.343**	26.23	0.00	-0.467**
LAmin	54.84	2.61	0.83	0.70†	0.102*	0.03	0.97†	-0,032
LA_10_	67.13	3.95	12.05	0.00	0.273**	6.12	0.00	-0.403**
LA_50_	61.77	3.16	5.88	0.00	0.205**	2.91	0.06†	-0.295*
LA_90_	57.95	2.83	2.53	0.00	0.134**	0.97	0.39†	-0,177

x̄: Average noise level.

***S*** standard deviation.

F statistic from the ANOVA. ***p*** statistical meaning for the variance (P-Value from the ANOVA).

SC Spearman correlation coefficient.

*p <0.05.

**p <0.01.

† means that there is no significant difference between the average of one level and another with a confidence level of 99%.

For the case of the ANOVA percentiles of the middle hours, a significant difference was seen for the hourly averages middle hours in the three parameters (LA_10_, LA_50_ and LA_90_) with a confidence level of 99% and it is ratified by the Spearman coefficient presented with the same level of meaning (Table 1). In terms of the averages by shift, ANOVA showed only significant differences for LA_10_, showing that the surrounding and average NICU noise was similar in terms of the shifts. This was confirmed with the Spearman analysis that maintained a significant and indirect relationship with LA_10_ and LA_50_ (Table 1).

Finally, the Spearman analysis showed that the noise results are consistent and coherent given that there is a direct and significant correlation between the LAeq-LA_50_, LAmax-LA_10_ and LAmin-LA_90_ variable pairs (Table 2).

**Table 2:** Correlation of the acoustic parameters in the NICU of the hospital.

Correlation	Hourly Integration	Shift Integration
LAeq versus LA_50_	0.954**	0.973**
LAmax versus LA_10_	0.890**	0.916**
LAmin versus LA_90_	0.833**	0.946**

**p<0.01

## Discussion

With the obtained results, there were greater records seen during the morning in contrast with those presented in the night, where the values are more constant. The difference can be attributed to medical controls, sampling, radiography, social work and family visits, which occur mostly in the daytime (morning and afternoon). This is confirmed by the hourly integrated averages that showed high values during times around the execution of the mentioned activities and being consequent with the behavior of the percentiles ((LA_10_, LA_50_ and LA_90_), which show a gradual decrease during night shifts. This dynamic has not only been reported for NICUs, but also for any type of ICU ^14^.

If the relationship between the two groups of acoustic parameters studied is analyzed in the two periods of time, it can be established that the maximum values (LAmax and LA_10_) and the noise level (LAeq and LA_50_) show a high variation compared to the minimum (LAmin) and bottom (LA_90_) values, which do not appear to be influenced by the routine events mentioned, such as feeding and family visits, among others, which is corroborated by the analysis of variance.

Comparing the noise levels reported by the study with the levels recorded by previously published studies, similar values are observed to those presented by different authors in neonatal intensive care units whose values fluctuate between 49-92 dB(A) ^6^ ^,^ ^8^ ^,^ ^10^ ^,^ ^13^ ^,^ ^15^ ^-^ ^19^; a range that includes the average values of LAmin and LAmax and even the average background noise level is greater than the minimum value of the range reported by the research.

The maximum noise levels suggested by the international standards for the protection against acoustic contamination collected by Garrido *et al*. ^20^, are exceeded by the results of this study, creating concern about the levels to which newborns and medical personnel are exposed. This situation gets even worse if the suggestion given by the Spanish Pediatrics Association (AEPED, for the Spanish original) is analyzed ^21^, which suggests that the background noise levels in the unit must not surpass 55 dB(A) and avoid surpassing 70 dB(A), a suggested value exceeded by the results.

These values are more inclusive than those recommended by Konkani and Oakley ^11^, who maintain that levels less than 40 dB(A) are required for the activities that require concentration, affirming that the greater values can cause interruptions. In the case of the care staff, it is a key limiting factor, given that the nurses must be capable of concentrating on caring for patients in order to avoid causing potential harm due to error ^3^ ^,^ ^11^.

On the other hand, when reviewing the results from a spectral footprint and the contribution of each noise band generated by the three monitors and telephone, in two of the four cases it was seen that the bands that contributed on a larger extent to the noise level correspond to the bands located in low frequencies; a result that contributes to increasing the risk of injuring the inner ear of the newborns. Studies show that low frequency sounds are more harmful to the ear's hair cells, causing microtears, vascular lesions and very frequently hearing loss ^22^ ^,^ ^23^. 

Various authors report in their studies values between 59-77 dB(A) for the vital sign monitors ^6^ ^,^ ^7^ ^,^ ^13^ ^,^ ^24^ ^,^ ^25^, coherent with the results obtained. For their part, the values obtained for the phone in the intensive care unit were less than those recorded by the literature ^7^ ^,^ ^13^ ^,^ ^24^. Notwithstanding, the presence of tones in the high frequencies can induce an abrupt response in the motor skills of the staff and newborns. Furthermore, if the average sound emission sources are compared to the suggestion made by the AEPED, the values exceed the level of 40 dB(A), suggested by this association ^21^.

The results obtained on the level of noise in the NICU show the need to design methods, strategies and/or programs according to the internal dynamic of the unit, which allow to decrease the noise records, and in this way, lower the risk of creating damaging environmental conditions for the recovery process of the newborns in the NICU. 

It is important to note that, among the study's limitations, the noise emission source measurements in the NICU were not possible to do in totally isolated conditions, due to the demand of the equipment and the dynamics of the unit; additionally, it is declared that it did not include a measuring protocol with a dosimeter in the methodology, the possible risk of exposure will be subject to the time of permanence of staff and/or newborns to the environmental noise levels reported by the sound level meter, which have been documented in other studies and discussed in this study. 

## Conclusions

In the NICU, a level of noise greater than the limits established by national and international groups was obtained. Also, there was evidence of an influence from the time of day and the shift in the acoustic parameters recorded in the unit. Finally, the sources identified and measured reported greater contributions in low frequencies, associated with a greater risk of harm for the newborn.
