# Supplementary material for: Noise level in a neonatal intensive care unit in Santa Marta - Colombia
Source: Colomb Med (Cali). 2017 Sep 30;48(3):120–5. doi: 10.25100/cm.v48i3.2173 (PMC5687863; doi:10.25100/cm.v48i3.2173)
Supplement: Translation

# Translation: Nivel de ruido de una unidad de cuidado intensivo neonatal en Santa Marta - Colombia

## Introducción 

En el ambiente de las unidades de cuidado intensivo se presentan diariamente estímulos auditivos para el neonato, asociados frecuentemente al ruido provocado por las alarmas de los equipos médicos, timbre de teléfonos, conversaciones del personal, cierre y apertura de puertas, caída de elementos dentro de la unidad ^1^.

Estos estímulos ocasionados por el ruido producen cuatro tipos de efectos adversos sobre los neonatos, especialmente en los prematuros como efectos somáticos, disturbios de sueño, daño auditivo y trastornos en el desarrollo emocional ^1^^,^^2^, además de las posibles repercusiones en el personal asistencial ^3^. Brown ^4^ afirmó que la estimulación auditiva en exceso crea respuestas fisiológicas negativas, como la apnea y las fluctuaciones en la frecuencia cardíaca, la presión arterial y saturación de oxígeno ^4^. Se estimó que el ruido de voces y alarmas de monitores pueden generar un aumento en el nivel de ruido cercano a los 120 decibelios A (dBA) en las unidades ^5^, provocando pérdida auditiva, alteraciones en el desarrollo del neonato, irritabilidad, estrés y efectos negativos sobre la formación del sistema nervioso sensorial del neonato ^6^.

Se han publicado estudios que muestran los niveles promedio de ruido en las unidades de cuidado intensivo neonatales (UCIN), en Chile se obtuvieron valores entre 45 - 80 dBA ^2^, en Lima (Perú) entre los 62-76 dBA ^7^^,^^8^, En Madrid y Huelva (España) entre 51-88 ^9^^,^^10^ Tabriz (Irán) entre 56-70 dBA ^6^, entre otros estudios que reportan entre 58-70 dBA ^8^. Valores altos si se comparan con los límites estipulados por la Organización Mundial de la Salud (OMS) que estableció valores de 35 dBA para el día y de 30 dBA para la noche ^11^. 

En la región del Caribe colombiano se adolece de estudios publicados relacionados con la temática que permitan verificar los niveles de ruido que se manejan en el interior de las UCIN de la región, razón por la que se evaluó en la ciudad de Santa Marta los niveles de ruido continuo equivalente en la unidad de cuidado neonatal de un Hospital.

## Materiales y Métodos

El estudio se desarrolló en la unidad de cuidado intensivo neonatal (UCIN) de un hospital ubicado en la ciudad de Santa Marta (Colombia), en un centro de atención médica de alta complejidad de la región, de carácter público que presta sus servicios médicos, al tiempo que se establece como escenario para la docencia universitaria en el área de la salud. 

La unidad cuenta con catorce camas habilitadas para el cuidado crítico del neonato; con una ocupación promedio una de 58% (8 ±1 una cama) y dispone de 5 ±1 personal asistencial ^12^. establecidos en tres turnos: mañana (7:00-13:00 h), tarde (13:00-19:00 h) y noche (19:00-7:00 h^+1)^. Así mismo existen 8 horarios de alimentación por día en lapso de 3 h iniciando a las 00:00 h, finalmente en la UCIN existen dos periodos de visitas de 20 min a las 11:00 h y 17:00 h

### Diseño del Muestreo

#### Nivel de ruido en la UCIN

El muestreo se realizó de forma continua durante 20 días en la UCIN, teniendo en cuenta la metodología expuesta en el estudio desarrollado por Vélez-Pereira AM ^13^ y Fortes-Garrido *et al* ^10^. Se empleó un sonómetro tipo I marca Casella modelo CEL-633-C1K1 y se programó para la toma de datos cada 60 segundos utilizando el filtro de ponderación frecuencial A y el filtro de ponderación temporal Fast. El sonómetro se ubicó en la unidad de cuidado intensivo teniendo en cuenta el estudio desarrollado por Vélez - Pereira ^13^, la dinámica interna de la UCI y condiciones brindadas por los coordinadores del personal asistencial. El punto de muestreo se ubicó en el ambiente de la unidad a una distancia de 60 cm del techo y de 215 cm de la pared.

Los parámetros acústicos registrados fueron el nivel continuo equivalente (LAeq), el nivel máximo (LAmax) y el nivel mínimo (LAmin) con el fin de analizar la variación temporal de ruido en la Unidad, adicionalmente se registraron los parámetros acústicos percentil 90 (LA_90_) para establecer el ruido de fondo de la unidad, el percentil 10 (LA_10_) para establecer la dinámica de los eventos pico o ruidos impulsivos y el percentil 50 (LA_50_) como un contraste del ruido en la unidad, el cual está asociado a la dinámica existente entre los valores picos y fondo en la unidad.

#### Fuentes de emisión de ruido

Se realizó una identificación visual de las posibles fuentes de emisión, y posteriormente se programó el sonómetro y se elaboró un análisis espectral en bandas de tercios de octava sin filtro de ponderación frecuencial (frecuencias planas) que permitiera determinar el aporte de cada banda a los niveles de ruido. La medición se realizó dentro de la unidad bajo la influencia de otras fuentes emisoras de ruido, justificando la inmovilidad de los equipos debido principalmente a la dinámica y demanda de cubículos en la unidad de cuidado intensivo. Las mediciones se realizaron a una distancia de 1.35 m de las fuentes y a 1.20 m sobre el nivel del suelo por espacio de tres minutos. Se utilizó el mismo sonómetro que se empleó para la medición del ruido ambiente de la UCIN.

### Procesamiento de la Información

#### Nivel continúo equivalente de ruido en la unidad

En Microsoft Excel (r) se realizó la integración de los datos registros de todos los parámetros de ruido bajo dos períodos, el primero correspondiente a los turnos del personal asistencial (mañana, tarde y noche) y el segundo correspondiente a los valores horarios. Estos periodos de integración se establecieron con el fin de determinar la variación horaria del ruido a largo de un día y de los turnos, permitiendo establecer la posible influencia en la dinámica del nivel de ruido en la UCIN.

Las integraciones se realizaron siguiendo la ecuación 1.


Ecuación 1


Donde *j* corresponde al parámetro acústico integrado, mientras que *i* corresponde al número de observaciones o mediciones realizadas en el intervalo de tiempo de integración (hora o turno) y n es el total de datos observado para el parámetro acústico *j* en el intervalo de tiempo *i*. 

### Análisis estadístico

El análisis estadístico se ejecutó con el programa IBM SPSS 20, se realizó una ANOVA ^6^^,^^13^ para comparar las medias de los diferentes tiempos de integración. Así mismo la información es ratificada mediante el análisis de correspondencia de Spearman. Finalmente se realizó un análisis de correspondencia entre los dos grupos de parámetros acústicos (LAeq vs LA_50_, LAmax vs LA_10_ y LAmin vs LA_90_), con el fin de verificar la fiabilidad de los registros obtenidos.

## Resultados

Los valores del LAeq horario presentaron valores medios comprendidos entre los 59.54±0.50 dBA para las 3:00 h y 65.27 ±0.46 dBA para las 8:00 h, mostrando un valor máximo de 69.96 dBA (14:00 h) y un mínimo de 57.80 dBA (3:00 h). En la Fig. 1 (superior) se observa la variación de las medias horarias del día. En ella se observa que en las primeras horas del día (23:00-5:00 h) los valores de nivel continuo de ruido equivalente fueron mucho menor a lo presentado en el resto.

**Figura 1:** Nivel promedio de ruido a lo largo de un día de muestreo para los parámetros acústicos. Arriba: LAeq, LAmax y LAmin; Abajo: percentiles LA_50_, LA_10_ y LA_90_.

Para el LAmax se reportaron valores en un rango de 67.22 ±3.01 dBA (3:00 h) y 77.32 ±1.52 dBA (7:00 h), con valor máximo de 83.70 dBA (23:00 h) y un mínimo de 59.73 dBA (3:00 h). Finalmente, para el caso de los LAmin, los valores variaron entre 53.16 ±2.45 dBA (3:00 h) y 55.08 ±1.88 dBA (8:00 h). Con un máximo y mínimo de 61.23 dBA y 49.83 dBA respectivamente. La dinámica de estos dos últimos parámetros fue similar a la LAeq (Fig. 1 superior); no obstante, se observó que la variación de los niveles de ruido horario fue ligeramente mayor en los valores de LAmax, seguido por LAeq y LAmin. 

Por otro lado, los niveles de ruido por turnos mostraron que el LAeq varió entre 64.73 ±1.43 dBA y 63.01 ±1.45 dBA, con máximos de 68.05 dBA y un mínimo de 60.27 dBA. Seguidamente para el caso de LAmax se presentaron valores en un rango de 74.71 ±1.46 dBA a 76.99 ±1.41 dBA, con máximos y mínimos de 80.57 dBA y 71.56 dBA, respectivamente. Finalmente, el LAmin varió entre 50.54 ±2.1 dBA y 54.65 ±1.7 dBA, con máximo de 59.58 dBA y un mínimo de 51.45 dBA. Los valores máximos de estos rangos en los tres parámetros se presentaron en el turno de la mañana, los mínimos en el turno de la noche, mientras que los máximos y mínimos absolutos se presentaron en el horario de la tarde, exceptuando el mínimo de LAmin que se reportó en el turno de la noche (Fig. 2 superior). Por lo tanto, se puede afirmar que los parámetros acústicos descendieron en la madrugada (turno nocturno) y ascendieron en el turno de la mañana y tarde. Esta dinámica fue similar a las medias horarias pero los cambios fueron menores (Fig. 1 superior).

**Figura 2:** Nivel promedio de ruido a por día de muestreo para los parámetros acústicos. Arriba: LAeq, LAmax y LAmin; Abajo: percentiles LA_50_, LA_10_ y LA_90_.

Los parámetros acústicos LA_10_, LA_50_ y LA_90_, mostraron que los promedios horarios del ruido pico (LA_10_) variaron entre 62.25 ±2.39 dBA (3:00 h) y 68.58 ±1.69 dBA (18:00 h), con un máximo de 73.87 dBA y un mínimo de 54.76 dBA. El LA_50_ varió entre 58.39 ±2.35 dBA a las 3:00 h y 62.85 ±2.02 dBA a las 8:00 h, con un máximo y un mínimo de 58.00 dBA y 51.69 dBA respectivamente. El ruido de fondo (LA_90_), varió menos que los dos parámetros anteriores (55.51 ±2.42 dBA a las 3:00 h - 58.74 ±1.87 dBA a las 8:00 h), con un máximo de 63.70 dBA y mínimo de 50.61 dBA (^Fig.1^ inferior). Las fluctuaciones horarias de los percentiles mostraron que desde las 22:00 h a 4:00 h, presentaron un descenso progresivo, el cual aumentó a las 5:00 h y permaneció estable el resto del día. En cuanto a las medias por turno (Fig. 2 inferior), se observó mayor variación en LA_10_ (rango 67.94 ±1.47 - 66.85 ±1.41 dBA, máximo 71.31 dBA, mínimo 63.06 dBA), seguido del LA_50_ (rango 62.30 ±1.53 - 61.00 ±1.18 dBA, máximo 65.91 dBA, mínimo 58.64 dBA), y finalizando LA_90_ como el parámetro más estable (rango 58.18 ±1.61 - 57.39 ±1.81 dBA, máximo 61.88 dBA, mínimo 54.36 dBA).

En cuanto a las fuentes de emisión, se lograron identificar y caracterizar 4 fuentes, tres de ellas fueron monitores de signos vitales de marca y modelos diferentes y el teléfono empleado para la comunicación (Fig. 3). En general los resultados manifestaron aportes similares en las diferentes bandas de tercio de octavas. Donde las alarmas de los monitores 1 y 3 tuvieron menor aporte en las frecuencias bajas (de 0 a 125 hz), mientras que la alarma del monitor 2 y el teléfono presentaron menor aporte en las frecuencias altas (mayores a 500 Hz).

**Figura 3:** Espectro fuentes de ruido estudiadas. Resultados del espectro de a) monitor de signos A1, b) monitor de signos A2, c) Monitor de signos A3, d) teléfono.

Puntualmente la alarma del monitor 1 varió entre 30.6-62.1 dB con una desviación estándar de 7.8 dB, presentó un mayor valor en las frecuencias altas con una media de 58.09 dB, seguida de las frecuencias medias (160-400 Hz) con una media de 52.9 dB y finalizando con las frecuencias bajas de 51.86 dB (Fig. 3a). Esta misma dinámica se presentó con la alarma del monitor 3 (Fig. 3c), con un rango de variación similar (29.3-62.7 dB, desviación estándar 7.7 dB), así como el aporte por frecuencias (frecuencias altas: 56.65 dB, medias: 56.34 dB, bajas: 50.46 dB). La alarma del segundo monitor (Fig. 3b) aportó más en las frecuencias bajas (50.55 dB), seguido de las altas (49.20 dB) y las medias (48.36 dB), variando en un rango de 17.7-56.6 dB y una desviación estándar de 8.8 dB. El espectro del teléfono presentó un orden de frecuencias por aporte de baja (57.92 dB), medias (43.79 dB) y altas (42.91 dB), con un rango de variación mayor (16.8-63.3 dB) y desviación estándar más alta (12.9 dB). En esta última fuente se presentaron unas tonalidades marcadas en tres bandas de tercio de octava de valor central de 1,250 y 2,500 Hz (Fig. 3D).

El análisis de varianza evidenció una diferencia significativa entre una media horaria y otra, con un nivel de significancia del 99% para LAeq y LAmax, mientras que para el LAmin no. En contraste con el ANOVA los coeficientes de Spearman ratificaron la información, mostrando una relación significativa con *p* <0.01 entre las medias horarias de los LAmax y LAeq, mientras que el LAmin presentó una relación débil con un *p* <0.05 (Tabla 1). Estos resultados fueron similares para las medias por turno, solo que coeficiente de correlación de Spearman estableció una relación indirecta y significativa para LAeq y LAmax, y no significativa para LAmin (Tabla 1).

Para el caso de los percentiles el ANOVA de las medias horarias mostró diferencia significativa para las medias horarias en los tres parámetros (LA_10_, LA_50_ y LA_90_) con un nivel de confianza del 99% y es ratificada por el coeficiente de Spearman presentado con el mismo nivel de significación (Tabla 1). En cuanto a las medias por turno, el ANOVA mostró solo diferencias significativas para el LA_10_, manifestando que el ruido de fondo y medio de la UCIN fue similar a lo largo de los turnos, esto se confirmó con el análisis de Spearman que manifestó una relación significativa e indirecta con el LA_10_ y LA_50_ (Tabla 1).

**Tabla 1:** Análisis de Varianza de los niveles de ruido en la UCIN del Hospital.

Parámetro Acústico	Estadístico		Integración Horaria			Integración por Turno		
	x̄	*S*	F	P**	CS	F	p**	CS
LAeq	64.00	3.62	10.94	0.00	0.269**	16.44	0.01	-0.381**
LAmax	76.04	5.73	25.04	0.00	0.343**	26.23	0.00	-0.467**
LAmin	54.84	2.61	0.83	0.70†	0.102*	0.03	0.97†	-0.032
LA_10_	67.13	3.95	12.05	0.00	0.273**	6.12	0.00	-0.403**
LA_50_	61.77	3.16	5.88	0.00	0.205**	2.91	0.06†	-0.295*
LA_90_	57.95	2.83	2.53	0.00	0.134**	0.97	0.39†	-0.177

x̄ Promedio del nivel de ruido.

***S*** desviación estándar.

F Razón o estadístico F de análisis ANOVA. ***p*** significancia estadística de la varianza (P-Valor del ANOVA).

CS Coeficiente de correlación de Spearman.

*p <0.05.

**p <0.01.

† indica que no existe una diferencia significativa entre una media de un nivel y otro con un nivel de confianza del 99%.

Finalmente, el análisis de Spearman evidenció que los resultados de ruido son consistentes y coherentes puesto que existe una correlación directa y significativa entre los pares de variables LAeq-LA_50_, LAmax-LA_10_ y LAmin-LA_90_ (Tabla 2).

**Tabla 2:** Correlación de los parámetros acústicos en la UCIN del Hospital.

Correlación	Integración Horaria	Integración por Turno
LAeq versus LA_50_	0.954**	0.973**
LAmax versus LA_10_	0.890**	0.916**
LAmin versus LA_90_	0.833**	0.946**

**p <0.01

## Discusión

Con los resultados obtenidos se evidencian mayores registros durante el período de la mañana en contraste con los presentados durante el período nocturno en donde los valores son más constantes, La diferencia se puede atribuir a la realización de controles médicos, toma de laboratorios, radiografías, visitas de trabajo social y familiares, que ocurren en su mayoría durante el horario diurno (mañana y tarde). Lo anterior se confirma por medio de las medias horarias integradas que presentaron valores altos durante horarios cercanos a la ejecución de las actividades mencionadas y resultando consecuente con el comportamiento de los percentiles (LA_10_, LA_50_ y LA_90_) que muestran un descenso gradual en el horario nocturno. Dinámica que no solo ha sido reportada en para las UCIN, sino para cualquier tipo de UCI ^14^.

Si se analiza la relación presentada entre los dos grupos de parámetros acústicos estudiados en los dos periodos de tiempo, se puede establecer que los valores máximos (LAmax y LA_10_) y el nivel de ruido (LAeq y LA_50_) presentan una variación alta en comparación con los valores mínimo (LAmin) y de Fondo (LA_90_) que parecen no estar influenciado por los eventos rutinarios mencionados como alimentación y visita de familiares entre otros, y lo cual es corroborado por el análisis de varianza.

Comparando los niveles de ruido reportados por el estudio realizado con los niveles registrados por estudios publicados previamente, se observan valores semejantes a los presentados por distintos autores en unidades de cuidado intensivo neonatal cuyos valores oscilan entre los 49-92 dBA ^6^^,^^8^^,^^10^^,^^13^^,^^15^^-^^19^. Rango en el que se incluyen los valores promedio del LAmin y LAmax, incluso el nivel de ruido de fondo promedio está por encima del valor mínimo del rango reportado por las investigaciones.

Los niveles máximos de ruido sugerido por los estándares internacionales para la protección contra la contaminación acustica recopilados por Garrido *et al*. ^20^, son superados por los resultados del presente estudio, generando preocupación sobre los niveles a los que están expuestos los neonatos y el personal médico. Esta situación es más agravante si se analiza la sugerencia dada por la Asociación Española de Pediatría AEPED ^21^, quien sugiere que los niveles de ruido de fondo en la unidad no deberán superar los 55 dBA y evitar superar los 70 dBA, valor de sugerencia superado por los resultados.

Estos valores son inclusive mayor a los recomendados por Konkani y Oakley ^11^, quienes manifiestan que se requieren niveles menores a 40 dBA para las actividades que requieren concentración, afirmando que valores superiores pueden causar interrupciones. Y para el caso del personal asistencial, es un factor limitante clave puesto que las enfermeras deben ser capaces de concentrarse en sus actividades de cuidado de los pacientes, para evitar inducir a un riesgo potencial para el error ^3^^,^^11^.

Por otro lado revisando los resultados de la huella espectral y la contribución de cada banda del ruido generado por los tres monitores y el teléfono, se encontró en dos de los cuatro casos que las bandas que aportaron en mayor medida al nivel de ruido corresponden a las bandas ubicadas en frecuencias bajas, resultado que contribuye a aumentar el riesgo de lesión del oído interno sobre los neonatos, estudios muestran que sonidos en frecuencias bajas son más perjudiciales para las células ciliadas del oído interno causando microdesgarros y lesiones vasculares, ocasionando con mayor frecuencia perdidas auditivas ^22^^,^^23^.

Varios autores reportan en sus estudios valores entre los 59-77 dBA para los monitores de signos vitales ^6^^,^^7^^,^^13^^,^^24^^,^^25^, coherentes con los resultados obtenidos, por su parte los valores obtenidos para el teléfono en la unidad de cuidado intensivo fueron menores a los registrados por la literatura ^7^^,^^13^^,^^24^. No obstante, la presencia de tonalidades en las frecuencias altas puede inducir a respuesta abruptas en la motricidad del personal y los neonatos. Así mismo, si se comparan las medias de las emisiones de ruido de las fuentes con la sugerencia planteada por la AEPED, los valores superan el nivel de 40 dBA que es sugerido por esta asociación ^21^. 

Los resultados obtenidos sobre el nivel de ruido en la UCIN, evidencia la necesidad de diseñar medidas, estrategias y/o programas acordes con la dinámica interna de la unidad que permita disminuir los registros de ruido, y de esta manera disminuir el riesgo en la generación de condiciones ambientales perjudiciales para el proceso de recuperación de los neonatos en la UCIN. 

Es importante indicar como limitaciones del estudio, que las mediciones de las fuentes de emisión de ruido en la UCIN, no fue posible realizarse en condiciones de total aislamiento, debido a la demanda de los equipos y dinámica de la unidad; adicionalmente se aclara que al no incluir en la metodología un protocolo de mediciones con dosímetro, el posible riesgo de exposición estará sujeto al tiempo de permanencia del personal y/o neonato a los niveles de ruido ambiental reportados por el sonómetro, los cuales que han sido documentado en otros estudios y discutidos en el presente estudio. 

## Conclusiones

En la UCIN se obtuvo un nivel de ruido superior a los límites establecidos por organismos nacionales e internacionales. También se evidenció una influencia de la hora del día y el turno en los parámetros acústicos registrados en la unidad. Finalmente, las fuentes identificadas y medidas reportaron mayores aportes en las frecuencias bajas, asociado a un mayor riesgo de daño en el neonato.
